# The Anti-Cancer Activity of Lycopene: A Systematic Review of Human and Animal Studies

**DOI:** 10.3390/nu14235152

**Published:** 2022-12-03

**Authors:** Aleksandra Kapała, Małgorzata Szlendak, Emilia Motacka

**Affiliations:** 1 Department of Clinical Nutrition, Maria Skłodowska-Curie National Research Institute of Oncology (MSCNRIO), 02-781 Warsaw, Poland; 2 Department of Oncology, Medical University of Warsaw, 02-091 Warsaw, Poland

**Keywords:** lycopene, cancer, in vivo study, nutraceuticals

## Abstract

Lycopene is a nutraceutical with health-promoting and anti-cancer activities, but due to a lack of evidence, there are no recommendations regarding its use and dosage. This review aimed to evaluate the benefits of lycopene supplementation in cancer prevention and treatment based on the results of in vivo studies. We identified 72 human and animal studies that were then analysed for endpoints such as cancer incidence, improvement in treatment outcomes, and the mechanisms of lycopene action. We concluded that the results of most of the reviewed in vivo studies confirmed the anti-cancer activities of lycopene. Most of the studies concerned prostate cancer, reflecting the number of in vitro studies. The reported mechanisms of lycopene action in vivo included regulation of oxidative and inflammatory processes, induction of apoptosis, and inhibition of cell division, angiogenesis, and metastasis formation. The predominance of particular mechanisms seemed to depend on tumour organ localisation and the local storage capacity of lycopene. Finally, there is a need to look for predictive factors to identify a population that may benefit from lycopene supplementation. The potential candidates appear to be race, single nucleotide polymorphisms in carotene-cleaving enzymes, some genetic abbreviations, and insulin-like growth factor-dependent and inflammatory diseases.

## 1. Introduction

Lycopene is a carotenoid nutraceutical that enhances protection against cancer, cardiovascular disease, hypertension, and neurodegenerative and inflammatory diseases [1]. It is naturally found mainly as the trans isomer in varying concentrations in tomatoes, but also in other red fruits and vegetables such as red carrots, watermelons, grapefruits, and papayas [2]. The lycopene content of tomatoes ranges from 0.88 to 7.74 mg/100 g and depends on the species and ripening stage [3]. Lycopene undergoes cis isomerisation under heat induction, which improves its bioavailability [4]. The accumulation of lycopene also depends on other factors, such as age, body weight, gastrointestinal status, lifestyle habits, or food intake [5]. Large molecules such as pectins hinder lycopene absorption, while fat facilitates it. Therefore, oil-resolved lycopene dietary supplements may be more efficient than food sources [6].

Lycopene has no pro-vitamin A activity, but its 11 conjugated double bonds are twice as numerous as the physical quenching rate constant with singlet oxygen compared to β-carotene [7], resulting in its protective role against reactions that initiate carcinogenesis. Our research provided ample evidence for the anti-cancer role of lycopene in the prostate and breast cancer cell lines. An interesting feature of lycopene is its dual effect on oxidative processes. In addition to the aforementioned antioxidant capacity, in the process of oncogenesis, lycopene may show an opposite pro-oxidative effect that supports the elimination of pathological cells. In in vitro studies, lycopene has shown anti-neoplastic activity by interfering with cancer cell migration, invasion, vessel formation, and microenvironment regulation, and by enhancing apoptosis [8].

Our research aimed to assess whether the successes of in vitro studies are reflected in human and animal studies where the final effect of lycopene depends on other factors, such as its absorption and distribution, the influence of the microenvironment, and drug interactions.

## 2. Methods

### 2.1. Eligibility Criteria and Search Strategy

**Figure 1:**
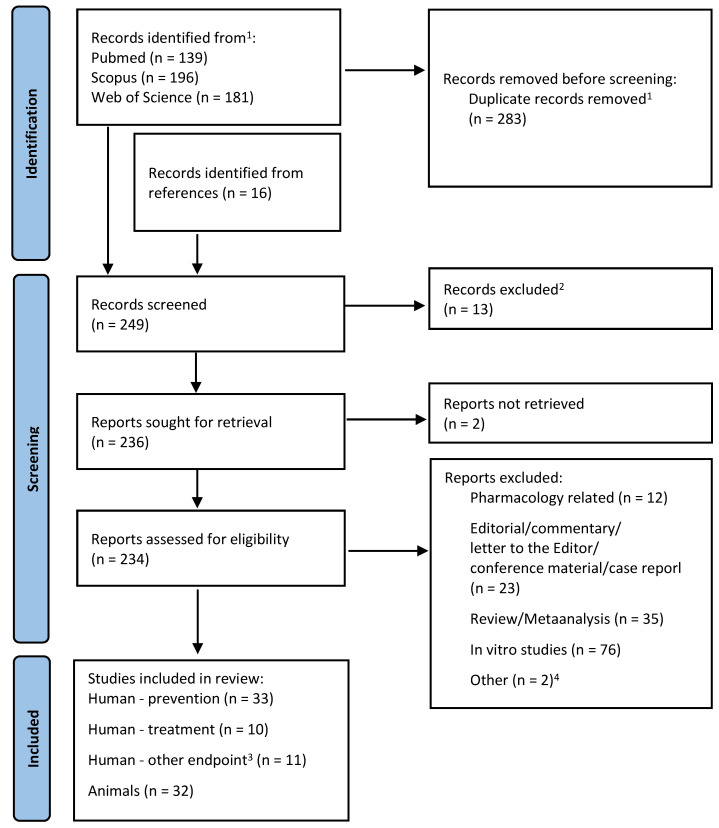
PRISMA flow diagram. ^1^ By automation tools based on PubMed ID (PMID). ^2^ Duplicates identified manually by title, DOI, or abstract. ^3^ Endpoint not related to cancer incidence, growth, or mortality. ^4^ One included preliminary results of the study that had been already included in the review and the second was an epidemiological study.

The PubMed, Scopus and Web of Science databases were comprehensively screened for papers about lycopene in cancer. The search keywords were “cancer” and “lycopene”, and results were limited to original papers published in English until June 2022. The search terms are shown in Supplement Table S1. The identification of human and animal studies was performed according to PRISMA guidelines [9]. After removing the duplicates, all the abstracts were screened to identify the original in vivo studies, which were further divided according to their endpoints, as shown in Figure 1. Studies related to pharmacology, pharmacodynamics, or pharmacokinetics were excluded, as well as editorial and conference materials, in vitro studies, and secondary analyses. The references of the latter were screened to identify the papers that met the inclusion criteria but were not found in the database search. Thus, such references were the secondary sources of papers.

### 2.2. Data Extraction

Data extraction was performed twice to avoid bias. The papers were divided into four subgroups depending on their content: human studies concerning cancer treatment, prevention, or with other endpoints that mostly concerned the mechanism of lycopene action, and animal research (Figure 1). The data collected from the papers included the size of the studied population, the type of study and intervention, the source and doses of supplemented lycopene, the exact endpoints, and the most important findings.

## 3. Results

**Figure 2:**
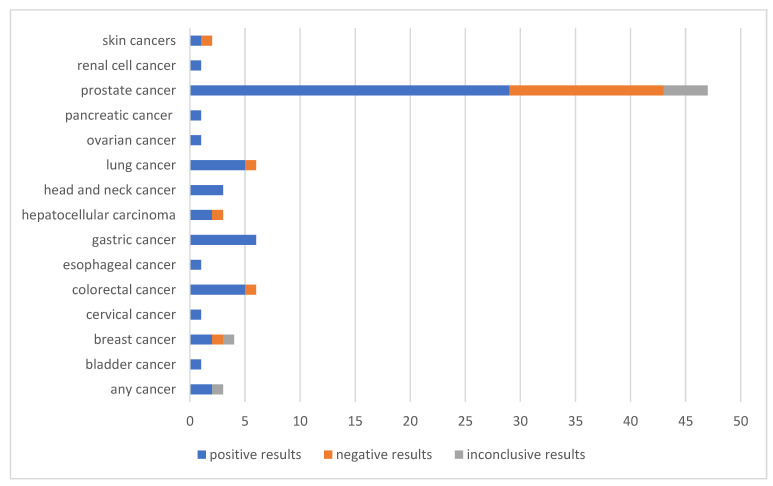
Outcomes of in vivo studies on the anti-cancer effect of lycopene.

The search algorithm that we used enabled us to identify 86 in vivo studies on lycopene in cancer (Figure 1). Of the papers, 47 were about prostate cancer [10,11,12,13,14,15,16,17,18,19,20,21,22,23,24,25,26,27,28,29,30,31,32,33,34,35,36,37,38,39,40,41,42,43,44,45,46,47,48,49,50,51,52,53,54,55,56], and 39 described the effects of lycopene on the development of other cancers [57,58,59,60,61,62,63,64,65,66,67,68,69,70,71,72,73,74,75,76,77,78,79,80,81,82,83,84,85,86,87,88,89,90,91,92,93,94,95]. About 70% of all the studies confirmed various anti-cancer properties of lycopene (Figure 2). A brief overview of all the analysed papers, including the data on their methods, population, endpoints, and results, can be found in the supplements divided according to the papers’ content: cancer prevention and treatment in humans, other research in humans, and animal research (Supplements Tables S2–5). The mechanisms that were studied and confirmed in the analysed in vivo studies are presented in Table 1.

### 3.1. Prostate Cancer

We found 20 papers on lycopene and the risk of prostate carcinogenesis in humans (Supplement Table S2). Most of them (n = 15) were observational studies in which the incidence of prostate cancer was assessed in relation to the declared amount of consumed products containing lycopene. The studies were retrospective and prospective, with a follow-up period of 6 months to 23 years. The antitumour activity of lycopene was confirmed in most studies (n = 11), in contrast to the intervention studies, in which four out of five did not demonstrate the antitumour activity of lycopene. Only one study indirectly concluded that the pyruvate-lowering effect of lycopene might be causally related to a reduced risk of prostate cancer [55].

The animal studies had inconclusive results regarding the preventive role of lycopene in prostate cancer (Supplement Table S4). In three studies, lycopene failed to reduce prostate carcinogenesis [16,35,39], but in two studies, lycopene was able to [15,34]. A fourfold reduction in the incidence of prostate cancer in mice was achieved when lycopene was supplemented with vitamin E and selenium [34].

The role of lycopene in cancer treatment in humans was studied only in prostate cancer (Supplement Table S3), and the benefit was confirmed by most of the studies (7 out of 10). It was shown that lycopene improved the treatment outcomes of locally advanced prostate cancer [25,49], reduced prostate cancer-specific mortality in men at high risk for prostate cancer [30], delayed the progression of recurrent prostate cancer regardless of hormone sensitivity status [19], improved the response to docetaxel chemotherapy in advanced castrate-resistant prostate cancer [33], and stabilised the effect of orchidectomy in a group of patients with advanced metastatic prostate cancer [51]. In addition, lycopene improved the quality of life, and provided relief from bone pain and control of lower urinary tract symptoms [50,51]. In some studies, lycopene brought no benefit in the treatment of patients in the early stages of prostate cancer recurrence (i.e., in biochemically relapsed prostate cancer [45]) or of patients with advanced hormone-refractory prostate cancer [52,53]. However, the latter population was included in three other studies that confirmed the positive role of lycopene in cancer treatment [19,33,50]. Moreover, the presented results did not ultimately show that lycopene has no role in cancer treatment, as one subgroup of patients had stable prostate-specific antigen (PSA), which was interpreted as a negative result consistent with the primary endpoint of PSA reduction, but stabilisation might have been the benefit brought by lycopene [48].

All six animal studies that examined the therapeutic effect of lycopene on prostate cancer obtained positive results and showed that supplementation caused tumour volume or weight reduction, apoptosis augmentation, prostate cancer-specific mortality decrease, or prostate cancer-free survival prolongation [22,27,36,54]. In one study, the therapeutic effect of lycopene was enhanced in combination with docetaxel, where an approximately 98% increase in apoptotic cells was achieved when lycopene + docetaxel was used compared to when docetaxel alone was used [22]. Lycopene co-supplementation with vitamin E also showed an improvement in the results of prostate cancer treatment, and in one of the studies the beneficial effect of lycopene was observed only in the case of such combined supplementation [28,36].

### 3.2. Other Cancers

#### 3.2.1. Positive Results That Confirm the Anti-Cancer Effect of Lycopene

The preventive potential of lycopene in female cancers was demonstrated in an animal model of ovarian cancer with predominant antioxidant and anti-inflammatory mechanisms [58]. In cervical cancer and cervical intra-epithelial neoplasia, the mean plasma levels of carotenoids—including lycopene—were significantly lowered compared to the healthy controls, which might indirectly indicate that antioxidant deficiency plays a role in cervical carcinogenesis [70]. In breast cancer, complex supplementation containing lycopene and other antioxidants reduced skin toxicity during radiotherapy used in radical treatment [75]. In addition, lycopene–genistein co-supplementation showed a protective effect against mammary carcinogenesis in an animal model [68].

With regard to cancers of the digestive system, lycopene intake showed a strong protective effect against stomach cancer, regardless of *H. pylori* status [82]. The mechanism of lycopene benefits in animal models of gastric and oesophageal cancer is based on modulation of carcinogen-induced tumour cell proliferation and apoptosis and enhancement of antioxidant and immune activities in the cancer microenvironment [71,72,73,81,83,93]. The conclusions about the role of lycopene in colorectal cancer were uncertain, but the growth of colon cancer in mice supplemented with lycopene alone or with lycopene and fish oil was inhibited [63]. Two independent studies confirmed the role of lycopene in inhibiting colorectal carcinogenesis via an insulin-like growth factor (IGF)-dependent mechanism [61,76]. Lycopene reduced skin toxicity during treatment with panitumumab in the mechanism of tissue protection from oxidative stress [90]. A lycopene-rich diet was shown to reduce the incidence of pancreatic cancer in humans by 31% [78]. In hepatocellular carcinoma mouse models, the benefit of lycopene was associated with attenuation of tumour invasion, proliferation, and angiogenesis, but this has not been confirmed in a rat model [60,62,88].

All the studies about lycopene in head and neck cancer confirmed its anti-cancer activity. The levels of lycopene were inversely associated with incidence and mortality in these cancers [64,67]. Another study identified a population of head and neck cancer survivors with high lycopene serum levels. The hallmark of this population was hypermethylation of mediators and signalling genes, which caused decreased oxidative damage and reduced T-cell activation and might have been the benefit of lycopene [66].

There are limited data on the role of lycopene in urological neoplasms other than prostate cancer. However, in bladder cancer the lycopene levels were shown to have been lowered at a borderline level of significance, which may suggest a causal relationship [92]. Similarly, in animal studies, lycopene supplementation attenuated renal cell carcinogenesis [58].

In the animal model, lycopene supplementation prevented type B ultraviolet-induced photocarcinogenesis, which was not shown in the human observational studies [86,87]. Likewise, only in the animal models did supplementation with lycopene alone or in combination with D-arginine prevent lung carcinogenesis [80,85,91]. In a lung cancer mouse model, lycopene improved the efficiency of anti-PD-1 therapy [79].

#### 3.2.2. Negative Results That Deny the Anti-Cancer Effect of Lycopene

Only 5 of the 39 studies about the anti-cancer role of lycopene in tumours other than prostate cancer had negative results. In humans, neither higher dietary levels nor higher plasma lycopene levels were shown to be associated with a reduced risk of breast cancer [59]. In addition, in humans, lycopene seemed not to have reduced the risk of colorectal, skin, or lung cancer. However, for lung cancer the trend was different depending on sex, with a statistically insignificant reduction of risk in males [74,87,95]. In a rat model of hepatocellular carcinoma (HCC), the long-term administration of lycopene did not reduce the risk of hepatocarcinogenesis [60].

**Table 1:** Mechanisms of lycopene antitumour activity identified in in vivo studies.

Mechanism	Pathway	Cancer	Model	Reference
Inhibition of angiogenesis	Lower degree of angiogenesis in the tumour	Prostate cancer	Human	[24]
	↓ vascular endothelial growth factor (VEGF)	Prostate cancer	Animal	[27]
	↓ VEGF and CD31	Hepatocellular cancer	Animal	[62,88]
Reduction of oxidative DNA damage	↓ oxidative injury (↓ HIF-1α, Cyr61, NDRG1, BNIP3, STAT2 mRNA); ↑ antioxidant enzyme activities (SOD, CAT and GPx)	Gastric cancer	Animal	[73,83]
	↑ GSH, GPx, GST and GR	Gastric cancer	Animal	[71,93]
	↑ activity of enzymic antioxidants (superoxide dismutase, catalase, and glutathione peroxidase) and reduced levels of nonenzymic antioxidants (glutathione, vitamins E and C)	Lung cancer	Animal	[80]
	↓ level of malondialdehyde, ↑ NRF2 and its major target protein (heme oxygenase 1)	Ovarian cancer	Animal	[94]
	↓ serum thiobarbituric acid-reactive substances	Any cancer	Human	[77]
	↑ serum protein thiol levels	Prostate cancer	Human	[46]
Reduction of inflammation	↓ COX-2 and PGE2	Colon cancer	Animal	[69]
	↓ NF-κB i COX-2	Oesophageal cancer	Animal	[81]
	↑IL-4 and IL-10; ↓ IL-6 and TNFα	Gastric cancer	Animal	[73,83]
	Hypermethylation of CD40LG and TEC	Head and neck cancer	Human	[66]
	↓ NF-κB	Ovarian cancer	Animal	[94]
Enhanced cytotoxicity	Hypomethylation of CD8A and ↑ CD8 and CD8+ T cells	Head and neck cancer	Human	[66]
Enhanced apoptosis	↑ PPARγ, caspase-3	Oesophageal cancer	Animal	[81]
	Accumulation of tumour cells in the G(0)/G(1) phase and further apoptosis	Prostate cancer	Animal	[22,56]
	↓ FOXO3a	Skin cancers	Animal	[86]
	↓ phosphorylation of BAD	Lung cancer	Animal	[91]
	↑ caspase-3, Bax1	Gastric cancer	Animal	[72]
Inhibition of proliferation: downregulation of insulin-like growth factor (IGF)	↓ IGF-1	Breast cancer	Human	[65]
	↓ IGF-1; ↑ IGF-BP	Colorectal cancer	Human	[61,76]
	↑ IGF-BP3	Lung cancer Prostate cancer	Animal	[91] [27]
Inhibition of cell cycle	↑ cell cycle inhibitors p21(CIP1/WAF1) and p27(Kip1); ↓ factors for DNA division (PCNA, β-catenin, cyclin D1, and *c*-Myc proteins)	Colon cancer	Animal	[63,69]
	↑ p27(Kip1)	Prostate cancer	Animal	[34]
	↓ STAT3 by inducing the protein inhibitor	Ovarian cancer	Animal	[94]
	↓ CDK2 and CDK4	Skin cancers	Animal	[86]
	↑ p21 and Bax1; ↓ factors for DNA division (cyclin 1, PCNA)	Gastric cancer	Animal	[72]
	↓ PCNA	Hepatocellular cancer	Animal	[62]
	↓ PCNA	Prostate cancer	Animal	[27,34]
Inhibition of hormone-dependent carcinogenesis	↓ steroid target genes (cystatin-related protein 1 and 2, prostatic spermine-binding protein, prostatic steroid-binding protein C1, C2, and C3 chain, probasin)	Prostate cancer	Animal	[36]
Inhibition of metastasis	↓ MMP-7 and MMP-9	Colon cancer	Animal	[69]
	↓ MMP-2 and MMP-9	Hepatocellular cancer	Animal	[62,88]

## 4. Discussion

The identified mechanisms of lycopene’s interference with carcinogenesis in vivo cover the whole range of intracellular processes and their impacts on the tumour microenvironment (Table 1), which generally confirms the results obtained in in vitro studies [96]. Nevertheless, the outcomes of specific pathway analyses are inconclusive and require some comment.

One of the debatable results seems to be the influence of lycopene on angiogenesis. A large study observed that dietary intake of lycopene was associated with a reduced risk of lethal prostate cancer and a lower degree of angiogenesis in the tumour [24]. In fact, in another study, the vascular endothelial growth factor (VEGF) concentrations were significantly lower in the sera of mice treated with lycopene than in the control group, and this trend continued over time but had no effect on proliferation or apoptosis, while in another study, it influenced the tumour volume [16,27]. In the HCC animal model, lycopene supplementation appeared to have reduced metastasis, which the authors interpreted as the effect of angiogenesis reduction [62]. Although these results are sometimes seemingly inconsistent, they show the effect of lycopene on different stages of angiogenesis. Vascular formation in tumour tissues is a process that develops over time and occurs in a phase of rapid growth under the influence of local destruction and hypoxia, which cause endothelial cells to release angiogenic stimulators [32]. Therefore, the mechanism of angiogenesis is more important in tumour development and metastasis than in tumour initiation, which explains the negative results of the mentioned study in mice. VEGF level testing is the simplest tool for assessing the process of angiogenesis, but its inhibition is also indirectly expressed by tumour size and metastasis reduction, which was observed in studies where lycopene was used in the treatment of prostate cancer [19,25,97]. However, these observations are insufficient to predict the effect of lycopene on angiogenesis in other cancers because lycopene is largely stored in the prostate, liver, testicles, adipose tissue, and adrenal glands [98]. Therefore, lycopene might have a greater impact on prostate cancer than on tumours in other locations where it is supplied only from serum. This relationship was somehow observed in a study on the development of prostate, lung, and colon cancer in a mouse model [99].

Another questionable result is the effect of lycopene on the IGF-mediated pathway. In a study about lycopene in prostate cancer treatment, the supplementation did not alter the IGF1 and IGFBP3 levels [97]. The levels of both proteins were lowered after prostatectomy regardless of lycopene supplementation, which was inconsistent with the hypothesis based on the observation that in in vitro and ex vivo studies, lycopene caused a decrease in IGF1 (with mitogenic and anti-apoptotic properties) and an increase in IGFBP3 (with an opposite effect) [100,101]. Other in vivo studies on prostate cancer in humans and animals also showed no effect of lycopene on IGF levels, and only one study in mice showed an increase in IGFBP3 [15,27,32]. Interestingly, in the female population with breast cancer and patients with colorectal cancer, lycopene appeared to have lowered the IGF levels, and in the latter, to have raised the IGF-BP levels [61,65,76]. The discrepancies in the results depending on the tumour organ localisation may indicate that the insulin pathway has less importance in the development of prostate cancer than in the development of breast and colorectal cancer, which is indirectly confirmed by the results of analyses of the role of the IGF axis in carcinogenesis [102,103,104]. However, the data from in vivo studies indicate the need for further research on the role of lycopene in tumours dependent on IGF stimulation, such as thyroid, breast, ovary, colon and lung cancer, as well as in gliomas and sarcomas [105].

Another issue worth commenting on is the anti-inflammatory effect of lycopene, which has been comprehensively discussed by some studies [106] and may be relevant in the prevention of inflammation-associated cancers, such as oesophageal adenocarcinoma arising from chronic duodenogastric-oesophageal reflux, *H. pylori*-induced gastric cancer, colitis-associated colorectal cancers, pancreatic cancer related to chronic pancreatitis, or hepatocellular carcinoma caused by chronic inflammation of the viral, toxic, metabolic, or autoimmune aetiology [107,108,109]. In the studies conducted to date, lycopene has shown protection against experimental models of oesophagitis, *H. pylori*-induced gastritis, inflammatory bowel diseases, toxin- and viral-induced hepatitis, and alcoholic and non-alcoholic fatty liver disease [110,111,112,113,114,115,116,117,118]. The results of the cited studies define the groups of patients who should be the first to be included in prospective interventional studies with the use of lycopene in the treatment of inflammation and the prevention of cancer. To date, the role of lycopene in preventing these inflammatory neoplasms has been studied only retrospectively in gastric and colorectal cancer, showing the benefits and non-benefits of lycopene [33,60], respectively. However, these studies concerned cancer incidence and did not distinguish subpopulations with chronic inflammation; thus, the case remains open and requires further research.

A very important aspect in the design of clinical trials with lycopene is appropriate population selection and attention to racial differences, which affect pharmacodynamics and are associated with different baseline levels of lycopene in populations with different diets [26]. The relationships between race, lycopene, and carcinogenesis have been studied only in prostate cancer. One study showed that a lycopene-rich diet could protect against prostate cancer only in non-Hispanic white men [12]. On the other hand, it is known that black men are twice as likely as white men to develop prostate cancer. Perhaps this population would benefit from prophylactic lycopene supplementation or from popularising a lycopene-rich diet [119]. However, lycopene supplementation in African Americans lowered the levels of oxidation markers in patients diagnosed with benign prostatic hyperplasia, but not to a statistically significant degree [43]. In addition, non-ethnic factors may influence the bioavailability of lycopene, such as age, body mass index, drug intake, alcohol consumption, or smoking status [5,120,121,122,123,124].

The population of patients who could benefit from the anti-cancer properties of lycopene can be found by identifying the predictive factors for lycopene supplementation. In one of the prospective studies about lycopene in prostate cancer, it was found that supplementation always raised the serum level of lycopene but did not always enhance the accumulation of lycopene in the prostate, and that not serum but low prostate lycopene levels were correlated with prostate cancer [40]. From this observation, it may be concluded that there are at least two populations of patients with prostate cancer, and between them, those with a preserved prostate storage capacity may benefit from the anti-cancer properties of lycopene. Another candidate for the role of predictive factors is single nucleotide polymorphisms in β-carotene oxygenase 1, associated with plasma lycopene responses to lycopene supplementation [13], which was independently confirmed by a large genome-wide association study [125]. Another potential predictive factor is TMPRSS2:ERG gene fusion, a common prostate cancer disorder [126]. TMPRSS2:ERG-positive disease appears to be particularly sensitive to the anti-cancer effects of tomato sauce [44]. There are also reports that the action of lycopene may be modified by the XRCC1 genotype, which is responsible for DNA repair [38,127].

The use of nutraceuticals and dietary supplements in the prevention and treatment of cancers is highly controversial due to the likely additive effect of many substances from the daily diet and the difficulty of designing selective studies identifying the specific substances and doses at which the nutraceuticals and dietary supplements exhibit anti-cancer effects. The lack of established daily references for lycopene consumption has resulted in various dietary supplements’ unregulated lycopene content. A European Food Safety Authority panel recommends a lycopene intake of 0.5 mg/kg body weight per day, at most 75 mg per day, but some reviews have shown that a dietary or formulated lycopene consumption of even up to 3 g per day is safe [96,128,129]. The lack of toxicity of high doses of lycopene may be due to the fact that lycopene absorption has an upper limit of saturated concentration and is not absorbed when the dose exceeds 60 mg [129].

In the cited intervention studies, lycopene was supplied as a dietary component (tomato paste) or as ready-made supplements available in the countries where the studies were conducted (see Supplements). None of the studies compared the anti-cancer effect depending on the form of lycopene supply. Ready-made supplements are based on lycopene isoforms with bioavailability comparable to tomato paste [130]. They are the most frequently chosen form of intervention, probably due to their easily quantifiable and repeatable doses. In contrast, interventions such as dietary advice or a lycopene-rich diet are not specific enough to be reliably analysed.

Lycopene has been reported to improve treatment outcomes in prostate cancer when used with docetaxel, soy isoflavones, and orchidectomy to treat advanced prostate cancer [19,33,36,51]. In animal models, lycopene reduced prostate cancer progression when given with docetaxel, vitamins E and B, and carotene, and reduced colon cancer progression when co-supplemented with fish oil [28,36,56,69]. In a lung cancer mouse model, lycopene improved the efficiency of anti-PD-1 therapy [79]. The cancer-protective effect of lycopene was enhanced by green tea, selenium, and vitamin E in the prostate, by genistein in the breast, by D-arginine in the lungs, and by S-allylcysteine in the stomach [10,34,68,80,93]. Therefore, to reliably assess the anti-cancer role of lycopene in prospective interventional studies, the researchers should carefully plan the methods that they would use for testing the intake, serum, and tissue levels not only of lycopene but also of other substances that may positively or negatively interfere with its activity.

To our knowledge, the present study is the only one to date that has comprehensively assessed the anti-tumour role of lycopene in humans and animals in a clinical setting. As a result, we confirmed that in vivo lycopene exhibits anti-cancer effects previously observed in in vitro studies. However, we are aware that the limitation of the presented analysis is its qualitative nature and the inclusion of studies regardless of the risk of bias. We have deliberately not limited this review to high-quality studies because the available data on intervention studies are too little and heterogeneous to be used in statistical analyses. Further population-specific studies are needed to quantify the anti-tumour role of lycopene.

## 5. Conclusions

Most in vivo studies have confirmed the anti-cancer activities of lycopene, particularly in prostate cancer. Human and animal studies have confirmed the influence of lycopene on some hallmarks of cancer, but the contributions of particular mechanisms seem to depend on the tumour organ localisation. The heterogeneity of the analysed interventions shows the need for systematised prospective studies to determine the appropriate recommended dose of lycopene in the daily diet and to search for predictive factors for the use of lycopene, particularly for oncological indications. 

## Supplementary Materials

The following supporting information can be downloaded at: https://www.mdpi.com/article/10.3390/nu14235152/s1, Table S1: Search terms; Table S2: Table: Overview of in vivo studies on the role of lycopene in cancer prevention; Table S3: Table: Overview of in vivo studies on the role of lycopene in cancer treatment; Table S4: Table: Overview of the analysed animal-based studies; Table S5: Table: Overview of human in vivo studies on lycopene in cancer with endpoints other than outcomes of treatment or prevention.
