# Supplementary material for: The Anti-Cancer Activity of Lycopene: A Systematic Review of Human and Animal Studies
Source: Nutrients. 2022 Dec 3;14(23):5152. doi: 10.3390/nu14235152 (PMC9741066; doi:10.3390/nu14235152)
Supplement: Supplementary file 1 [file nutrients-14-05152-s001.zip › Supplement 1.pdf]

Supplement 1. Search terms

Pubmed

((((lycopene[Title]) AND (cancer[Title]))) NOT ('Meta-analysis'[Publication Type] OR review[Publication Type] OR 'systematic review'[Publication Type])) NOT ('Meta-analysis'[Title] OR Review'[Title] OR 'Systematic review'[Title]) Filters: Full text, Humans

and for animal research

((((lycopene[Title]) AND (cancer[Title]))) NOT ('Meta-analysis'[Publication Type] OR review[Publication Type] OR 'systematic review'[Publication Type])) NOT ('Meta-analysis'[Title] OR Review'[Title] OR 'Systematic review'[Title]) Filters: Other Animals

Scopus

TITLE(lycopene AND cancer) AND (LIMIT-TO (DOCTYPE,"ar") OR LIMIT-TO (DOCTYPE,"cp")) AND (LIMIT-TO (LANGUAGE,"English"))

Web of science

cancer AND lycopene (Title) and Article OR Data Paper (Document Type) not Database Review OR Editorial Material OR Review (Document Type) and English (Language)
