# Supplementary material for: The Anti-Cancer Activity of Lycopene: A Systematic Review of Human and Animal Studies
Source: Nutrients. 2022 Dec 3;14(23):5152. doi: 10.3390/nu14235152 (PMC9741066; doi:10.3390/nu14235152)
Supplement: Supplementary file 1 [file nutrients-14-05152-s001.zip › Supplement 2.pdf]

Supplement 2. Overview of human studies on the role of lycopene in cancer prevention

Reference	Cancer	Year	Country	Study design	Population size	Endpoint	Outcome	Finding
[84]	Any cancer	2020	USA	Observation	22,835	Cancer mortality	Positive	The beneficial effect of lycopene was confirmed regardless of age or body mass.
[92]	Bladder cancer	1989	USA	Observation	25,802	Retrospective incidence of bladder cancer in 12-year follow-up	Positive	The lycopene level was lower among the cases with cancers at a borderline level of significance.
[59]	Breast cancer	2005	USA	Observation	1,076	Incidence of breast cancer in women without prevalent cancer in 9.9-year follow-up	Negative	Neither higher dietary nor plasma lycopene levels were associated with a reduced risk of breast cancer.
[70]	Cervical cancer	1996	USA	Observation	235	Retrospective incidence of cervical cancer	Positive	The mean plasma levels of carotenoids (beta-carotene, lycopene and canthaxanthin) and alpha-tocopherol were significantly lower in women with cervical intra-epithelial neoplasia and cervical cancer.
[74]	Colorectal cancer	2006	Australia	Observation	1,442	Retrospective incidence of colorectal cancer	Negative	Lycopene was not associated with colorectal cancer risk.
[8[i]9]	Different cancers, including prostate cancer	2009	Finland	Observation	997	Incidence of any cancer in men in 12.6-year follow-up	Inconclusive	The higher circulating concentrations of lycopene may contribute to the lower risk of cancer, with the exception of prostate cancer.
[82]	Gastric cancer	2022	Vietnam	Observation	226	Retrospective incidence of stomach cancer	Positive	Both trans-lycopene and β-cryptoxanthin intake showed a strong protective effect against stomach cancer regardless of *H. pylori* status.
[66]	Head and neck cancer	2020	USA	Observation	23	Features of head and neck cancer survivors: DNA methylation profiles in leukocytes; physiological, clinical and lifestyle parameters related to inflammation (including lycopene levels)	Positive	The group of head and neck cancer survivors with high circulating lycopene levels also had significant differential methylation of transcriptional and translational regulators and genes in the T-cell receptor signalling pathway, including hypermethylation of the CD40 ligand (CD40LG) and Tec protein tyrosine kinase and hypomethylation of CD8A.

[95]	Lung cancer	2009	USA	Observation	77,126	Retrospective incidence of lung cancer	Negative	Lycopene supplement use was not associated with lung cancer risk. The use of individual lycopene supplements was associated with a 71% reduced risk for men but a 74% higher risk for women, but these associations were not statistically significant.
[87]	Melanoma, basal cell and squamous cell cancer	1995	USA	Observation	297	Retrospective incidence of skin cancer	Negative	There were no significant associations between serum micronutrient levels and the risk of subsequent skin cancer.
[64]	Oral cavity, pharynx or larynx	2004	USA	Observation	259	Cancer mortality	Positive	Lycopene was inversely associated with mortality in oral cavity, pharynx or larynx cancer.
[78]	Pancreatic cancer	2005	Canada	Observation	5,183	Retrospective incidence of prostate cancer	Positive	Lycopene, provided mainly by tomatoes, was associated with a 31% reduction in pancreatic cancer risk among men.
[23]	Prostate cancer	2017	Italy	Intervention: lycopene–selenium supplementation	209	Incidence of prostate cancer in patients with lower urinary tract symptoms or benign prostatic hyperplasia in 2-year follow-up	Negative	Selenium and lycopene supplementation does not influence prostate cancer incidence.
[21]	Prostate cancer	2020	USA	Observation	27,934	Incidence of prostate cancer in men without prevalent cancer in 7.9-year follow-up	Positive	Consumption of canned and cooked tomatoes may reduce the risk of prostate cancer.
[55]	Prostate cancer	2019	UK	Intervention: lycopene or lycopene-rich diet supplementation	72,729	159 serum metabolite measures and retrospective incidence of prostate cancer	Positive	Lycopene lowered the levels of pyruvate, which the Mendelian randomisation analysis suggests may be causally related to reduced prostate cancer risk.
[17]	Prostate cancer	2011	USA	Observation	3,434	Incidence of prostate cancer in men without prevalent cancer in 7-year follow-up	Negative	Lycopene intake does not affect prostate cancer risk.
[44]	Prostate cancer	2016	USA	Observation	46,719	Incidence of prostate cancer in men without prevalent cancer in 23-year follow-up	Positive	Tomato sauce consumption may play a role in reducing prostate cancers that harbour TMPRSS2:ERG.

[12]	Prostate cancer	2021	USA	Observation	637	Retrospective incidence of prostate cancer defined as total prostate-specific antigen (PSA) level and the ratio of free PSA	Positive	Sufficient lycopene intake from daily food could serve as a protector against prostate cancer, but such an association was observed only in non-Hispanic white men.
[11]	Prostate cancer	2005	China	Observation	404	Retrospective incidence of prostate cancer	Positive	The prostate cancer risk declined with increasing consumptions of lycopene, α-carotene, β-carotene, β-cryptoxanthin, lutein and zeaxanthin.
[24]	Prostate cancer	2014	USA	Observation	49,898	Retrospective incidence of prostate cancer	Positive	Dietary intake of lycopene was associated with reduced risk of lethal prostate cancer and with a lesser degree of angiogenesis in the tumour.
[10]	Prostate cancer	2007	China	Observation	404	Retrospective incidence of prostate cancer	Positive	Intake of green tea and lycopene reduced the risk of prostate cancer in Chinese men. The two substances have a stronger preventive effect together than separately.
[53]	Prostate cancer	2006	USA	Observation	29,361	Incidence of prostate cancer in men without prevalent cancer in 4.2-year follow-up	Negative	Greater lycopene or tomato product consumption does not protect from prostate cancer.
[26]	Prostate cancer	2002	USA	Observation	437	Retrospective incidence of prostate cancer	Positive	Lycopene was inversely associated with prostate cancer risk, particularly for aggressive disease. Serum lycopene concentrations were significantly lower in blacks than in whites.
[46]	Prostate cancer	1999	Canada	Observation	24	Retrospective incidence of prostate cancer	Positive	Serum protein thiol levels, which reflect DNA repair capacity, were significantly lower among the cancer patients with lower lycopene levels.
[41]	Prostate cancer	2010	Australia	Intervention: dietary advice and vitamin A supplementation (either synthetic beta-carotene or retinol)	321	Incidence of prostate cancer in 14-year follow-up	Negative	The serum concentrations of folate, lycopene, β-carotene and vitamins A and E had no associations with subsequent development of prostate cancer.
[47]	Prostate cancer	2002	USA	Observation	47,365	Retrospective incidence of prostate cancer	Positive	Frequent consumption of tomato products was associated with a lower risk of prostate cancer.
[18]	Prostate cancer	2007	USA	Observation	692	Retrospective incidence of prostate cancer	Negative	Lycopene and other carotenoids were unrelated to prostate cancer.

[37]	Prostate cancer	2001	USA	Observation	197	Retrospective incidence of prostate cancer	Positive	The study confirmed the inverse associations between lycopene, other carotenoids (e.g. zeaxanthin, lutein and beta-cryptoxanthin) and prostate cancer.
[20]	Prostate cancer	1999	USA	Observation	1,872	Incidence of prostate cancer in healthy men in 13-year follow-up	Positive	The results confirmed the inverse association of lycopene and prostate cancer, particularly for aggressive cancer and for men not consuming beta-carotene supplements.
[40]	Prostate cancer	2014	Italy	Intervention: 20–25 mg/day of lycopene for 6 months	32	Progression of high-grade prostatic intra-epithelial neoplasia to prostate cancer in 6-month follow-up	Negative	The study showed no overall benefits from lycopene supplementation.
[31]	Prostate cancer	2007	USA	Intervention: 30 mg/day of lycopene for 4 months	81	Serum PSA and lycopene	Negative	The lycopene-induced PSA lowering was transient.
[38]	Prostate cancer	2006	USA	Observation	251	Retrospective incidence of prostate cancer depending on the XRCC1 genotype, lycopene intake and plasma alpha-tocopherol and beta-carotene levels	Inconclusive	The association between lycopene and prostate cancer is complex and may be modified by other antioxidants and by the XRCC1 genotype (DNA repair genes).
[67]	Upper aerodigestive tract (oral cavity, pharynx, larynx, oesophageal cancer)	2000	Uruguay	Observation	729	Retrospective incidence of upper aerodigestive tract cancers	Positive	The food group composed of raw tomato and tomato-rich foods and lycopene itself showed a strong inverse association with upper aerodigestive tract cancers.

Reference:

[10] Jian, L.; Lee, A.H.; Binns, C.W. Tea and lycopene protect against prostate cancer. *Asia Pac. J. Clin. Nutr.* **2007**, *16* (Suppl. 1), 453–457.

[11] Jian, L.; Du, C.-J.; Lee, A.H.; Binns, C.W. Do dietary lycopene and other carotenoids protect against prostate cancer? *Int. J. Cancer* **2005**, *113*, 1010–1014. https://doi.org/10.1002/ijc.20667.

[12] Tang, L.; Jin, T.; Zeng, X.; Wang, J.-S. Lycopene inhibits the growth of human androgen-independent prostate cancer cells in vitro and in BALB/c nude mice. *J. Nutr.* **2005**, *135*, 287–290. https://doi.org/10.1093/jn/135.2.287.

[17] Canene-Adams, K.; Lindshield, B.L.; Wang, S.; Jeffery, E.H.; Clinton, S.K.; Erdman, J.W.J. Combinations of tomato and broccoli enhance antitumor activity in dunning r3327-h prostate adenocarcinomas. *Cancer Res.* **2007**, *67*, 836–843. https://doi.org/10.1158/0008-5472.CAN-06-3462.

[18] Beynon, R.A.; Richmond, R.C.; Santos Ferreira, D.L.; Ness, A.R.; May, M.; Smith, G.D.; Vincent, E.E.; Adams, C.; Ala-Korpela, M.; Würtz, P.; et al. Investigating the effects of lycopene and green tea on the metabolome of men at risk of prostate cancer: The ProDiet randomised controlled trial. *Int. J. Cancer* **2019**, *144*, 1918–1928. https://doi.org/10.1002/ijc.31929.
[20] Lu, Y.; Edwards, A.; Chen, Z.; Tseng, T.-S.; Li, M.; Gonzalez, G.V.; Zhang, K. Insufficient Lycopene Intake Is Associated With High Risk of Prostate Cancer: A Cross-Sectional Study From the National Health and Nutrition Examination Survey (2003–2010). *Front. Public Heal.* **2021**, *9*, 792572. https://doi.org/10.3389/fpubh.2021.792572.
[21] Moran, N.E.; Thomas-Ahner, J.M.; Fleming, J.L.; McElroy, J.P.; Mehl, R.; Grainger, E.M.; Riedl, K.M.; Toland, A.E.; Schwartz, S.J.; Clinton, S.K. Single Nucleotide Polymorphisms in β-Carotene Oxygenase 1 are Associated with Plasma Lycopene Responses to a Tomato-Soy Juice Intervention in Men with Prostate Cancer. *J. Nutr.* **2019**, *149*, 381–397. https://doi.org/10.1093/jn/nxy304.
[23] Konijeti, R.; Henning, S.; Moro, A.; Sheikh, A.; Elashoff, D.; Shapiro, A.; Ku, M.; Said, J.W.; Heber, D.; Cohen, P.; et al. Chemoprevention of prostate cancer with lycopene in the TRAMP model. *Prostate* **2010**, *70*, 1547–1554. https://doi.org/10.1002/pros.21190.
[24] Conlon, L.E.; Wallig, M.A.; Erdman, J.W.J. Low-lycopene containing tomato powder diet does not protect against prostate cancer in TRAMP mice. *Nutr. Res.* **2015**, *35*, 882–890. https://doi.org/10.1016/j.nutres.2015.07.003.
[26] Peters, U.; Leitzmann, M.F.; Chatterjee, N.; Wang, Y.; Albanes, D.; Gelmann, E.P.; Friesen, M.D.; Riboli, E.; Hayes, R.B. Serum lycopene, other carotenoids, and prostate cancer risk: A nested case-control study in the prostate, lung, colorectal, and ovarian cancer screening trial. *Cancer Epidemiol. Biomark. Prev.* **2007**, *16*, 962–968. https://doi.org/10.1158/1055-9965.EPI-06-0861.
[31] Zu, K.; Mucci, L.; Rosner, B.A.; Clinton, S.K.; Loda, M.; Stampfer, M.J.; Giovannucci, E. Dietary lycopene, angiogenesis, and prostate cancer: A prospective study in the prostate-specific antigen era. *J. Natl. Cancer Inst.* **2014**, *106*, djt430. https://doi.org/10.1093/jnci/djt430.
[37] Wang, Y.; Jacobs, E.J.; Newton, C.C.; McCullough, M.L. Lycopene, tomato products and prostate cancer-specific mortality among men diagnosed with nonmetastatic prostate cancer in the Cancer Prevention Study II Nutrition Cohort. *Int. J. Cancer* **2016**, *138*, 2846–2855. https://doi.org/10.1002/ijc.30027.
[38] Bunker, C.H.; McDonald, A.C.; Evans, R.W.; de la Rosa, N.; Boumosleh, J.M.; Patrick, A.L. A randomized trial of lycopene supplementation in Tobago men with high prostate cancer risk. *Nutr. Cancer* **2007**, *57*, 130–137. https://doi.org/10.1080/01635580701274046.
[40] Venkateswaran, V.; Fleshner, N.E.; Sugar, L.M.; Klotz, L.H. Antioxidants block prostate cancer in lady transgenic mice. *Cancer Res.* **2004**, *64*, 5891–5896. https://doi.org/10.1158/0008-5472.CAN-04-0690.
[41] Imaida, K.; Tamano, S.; Kato, K.; Ikeda, Y.; Asamoto, M.; Takahashi, S.; Nir, Z.; Murakoshi, M.; Nishino, H.; Shirai, T. Lack of chemopreventive effects of lycopene and curcumin on experimental rat prostate carcinogenesis. *Carcinogenesis* **2001**, *22*, 467–472. https://doi.org/10.1093/carcin/22.3.467.
[44] Goodman, M.; Bostick, R.M.; Ward, K.C.; Terry, P.D.; van Gils, C.H.; Taylor, J.A.; Mandel, J.S. Lycopene intake and prostate cancer risk: Effect modification by plasma antioxidants and the XRCC1 genotype. *Nutr. Cancer* **2006**, *55*, 13–20. https://doi.org/10.1207/s15327914nc5501_2.
[46] Mariani, S.; Lionetto, L.; Cavallari, M.; Tubaro, A.; Rasio, D.; De Nunzio, C.; Hong, G.M.; Borro, M.; Simmaco, M. Low prostate concentration of lycopene is associated with development of prostate cancer in patients with high-grade prostatic intraepithelial neoplasia. *Int. J. Mol. Sci.* **2014**, *15*, 1433–1440. https://doi.org/10.3390/ijms15011433.
[47] Beilby, J.; Ambrosini, G.L.; Rossi, E.; de Klerk, N.H.; Musk, A.W. Serum levels of folate, lycopene, β-carotene, retinol and vitamin E and prostate cancer risk. *Eur. J. Clin. Nutr.* **2010**, *64*, 1235–1238. https://doi.org/10.1038/ejcn.2010.124.
[53] Jatoi, A.; Burch, P.; Hillman, D.; Vanyo, J.M.; Dakhil, S.; Nikcevich, D.; Rowland, K.; Morton, R.; Flynn, P.J.; Young, C.; et al. A Tomato-Based, Lycopene-Containing Intervention for Androgen-Independent Prostate Cancer: Results of a Phase II Study from The North Central Cancer Treatment Group. *Urology* **2007**, *69*, 289–294. https://doi.org/10.1016/j.urology.2006.10.019.
[55] Ansari, M.S.; Gupta, N.P. Lycopene: A novel drug therapy in hormone refractory metastatic prostate cancer. *Urol. Oncol.* **2004**, *22*, 415–420. https://doi.org/10.1016/j.urolonc.2004.05.009.
[59] Sesso, H.D.; Buring, J.E.; Zhang, S.M.; Norkus, E.P.; Gaziano, J.M. Dietary and plasma lycopene and the risk of breast cancer. *Cancer Epidemiol. Biomark. Prev.* **2005**, *14*, 1074–1081. https://doi.org/10.1158/1055-9965.EPI-04-0683.

[64] Mayne, S.T.; Cartmel, B.; Lin, H.; Zheng, T.; Goodwin, W.J.J. Low plasma lycopene concentration is associated with increased mortality in a cohort of patients with prior oral, pharynx or larynx cancers. *J. Am. Coll. Nutr.* **2004**, *23*, 34–42. https://doi.org/10.1080/07315724.2004.10719340.
[66] Moody, L.; Crowder, S.L.; Fruge, A.D.; Locher, J.L.; Demark-Wahnefried, W.; Rogers, L.Q.; Delk-Licata, A.; Carroll, W.R.; Spencer, S.A.; Black, M.; et al. Epigenetic stratification of head and neck cancer survivors reveals differences in lycopene levels, alcohol consumption, and methylation of immune regulatory genes. *Clin. Epigenetics* **2020**, *12*, 138. https://doi.org/10.1186/s13148-020-00930-5.
[67] De Stefani, E.; Oreggia, F.; Boffetta, P.; Deneo-Pellegrini, H.; Ronco, A.; Mendilaharsu, M. Tomatoes, tomato-rich foods, lycopene and cancer of the upper aerodigestive tract: A case-control in Uruguay. *Oral Oncol.* **2000**, *36*, 47–53. https://doi.org/10.1016/s1368-8375(99)00050-0.
[70] Palan, P.R.; Mikhail, M.S.; Goldberg, G.L.; Basu, J.; Runowicz, C.D.; Romney, S.L. Plasma levels of beta-carotene, lycopene, canthaxanthin, retinol, and alpha- and tau-tocopherol in cervical intraepithelial neoplasia and cancer. *Clin. Cancer Res.* **1996**, *2*, 181–185.
[74] Kune, G.; Watson, L. Colorectal cancer protective effects and the dietary micronutrients folate, methionine, vitamins B6, B12, C, E, selenium, and lycopene. *Nutr. Cancer* **2006**, *56*, 11–21. https://doi.org/10.1207/s15327914nc5601_3.
[78] Nkondjock, A.; Ghadirian, P.; Johnson, K.C.; Krewski, D. Dietary intake of lycopene is associated with reduced pancreatic cancer risk. *J. Nutr.* **2005**, *135*, 592–597. https://doi.org/10.1093/jn/135.3.592.
[82] Sengngam, K.; Hoc, T.H.; Hang, D.V.; Tran Ngoan, L. Trans-Lycopene and β-Cryptoxanthin Intake and Stomach Cancer in Vietnamese Men: A Pilot Case-Control Study. *Asian Pac. J. Cancer Prev.* **2022**, *23*, 861–865. https://doi.org/10.31557/APJCP.2022.23.3.861
[84] Mazidi, M.; Ferns, G.A.; Banach, M. A high consumption of tomato and lycopene is associated with a lower risk of cancer mortality: Results from a multi-ethnic cohort. *Public Health Nutr.* **2020**, *23*, 1569–1575. https://doi.org/10.1017/S1368980019003227.
[87] Breslow, R.A.; Alberg, A.J.; Helzlsouer, K.J.; Bush, T.L.; Norkus, E.P.; Morris, J.S.; Spate, V.E.; Comstock, G.W. Serological precursors of cancer: Malignant melanoma, basal and squamous cell skin cancer, and prediagnostic levels of retinol, beta- carotene, lycopene, alpha-tocopherol, and selenium. *Cancer Epidemiol. Biomark. Prev.* **1995**, *4*, 837–842.
[89] Karppi, J.; Kurl, S.; Nurmi, T.; Rissanen, T.H.; Pukkala, E.; Nyyssönen, K. Serum lycopene and the risk of cancer: The Kuopio Ischaemic Heart Disease Risk Factor (KIHD) study. *Ann. Epidemiol.* **2009**, *19*, 512–518. https://doi.org/10.1016/j.annepidem.2009.03.017.
[92] Helzlsouer, K.J.; Comstock, G.W.; Morris, J.S. Selenium, lycopene, alpha-tocopherol, beta-carotene, retinol, and subsequent bladder cancer. *Cancer Res.* **1989**, *49*, 6144–6148.
[95] Satia, J.A.; Littman, A.; Slatore, C.G.; Galanko, J.A.; White, E. Long-term use of beta-carotene, retinol, lycopene, and lutein supplements and lung cancer risk: Results from the VITamins And Lifestyle (VITAL) study. *Am. J. Epidemiol.* **2009**, *169*, 815–828. https://doi.org/10.1093/aje/kwn409.
