# Supplementary material for: The Anti-Cancer Activity of Lycopene: A Systematic Review of Human and Animal Studies
Source: Nutrients. 2022 Dec 3;14(23):5152. doi: 10.3390/nu14235152 (PMC9741066; doi:10.3390/nu14235152)
Supplement: Supplementary file 1 [file nutrients-14-05152-s001.zip › Supplement 3.pdf]

Supplement 3. Overview of human studies on the role of lycopene in cancer treatment

Reference in paper	Cancer	Year	Country	Study design	Population size (with controls)	Lycopene supplementation	Endpoint	Outcome	Finding
[19]	Prostate cancer	2007	USA	Intervention	71	15 mg twice daily +/- 40 mg soy isoflavone mixture	Prostate-specific antigen (PSA) response	Positive	Lycopene and soy isoflavones have activities in prostate cancer patients with PSA relapse disease and may delay the progression of both hormone-refractory and hormone-sensitive prostate cancer.
[30]	Prostate cancer	2016	USA	Observation	14541	X	Prostate cancer-specific mortality	Positive	Among men with a high risk for cancers (T3-T4 or Gleason score 8–10, or nodal involvement), lycopene intake above median was associated with lower prostate cancer-specific mortality compared to intake below median.
[33]	Prostate cancer	2021	USA	Intervention	13	30 mg once daily + docetaxel 75 mg/m^2 every 21 days	PSA response, median time to PSA progression, duration of response and overall survival	Positive	A PSA response rate of 76.9% and median survival of 35.1 months compare favourably to the 45% PSA response rate and 17.4 months median survival reported for the 237 TAX trialists.
[51]	Prostate cancer	2003	India	Intervention	54	2 mg twice daily + orchidectomy	PSA response, response in bones and improvement in peak flow rate	Positive	Adding lycopene to orchidectomy produced a more reliable and consistent decrease in serum PSA level, primary and secondary tumour shrinkage, better bone pain relief, lower urinary tract symptoms and improved survival compared with orchidectomy alone.
[50]	Prostate cancer	2004	India	Intervention	20	10 mg once daily	PSA response, bone pain relief and ECOG (Eastern Cooperative Oncology Group) performance status	Positive	Lycopene supplementation reduced PSA, improved the ECOG performance status and bone pain relief and alleviated the lower urinary track symptoms.
[49]	Prostate cancer	2002	USA	Intervention	26	30 mg once daily	PSA response, tumour stage and extent of high-grade prostatic intra-epithelial neoplasia (HGPIN)	Positive	The intervention group had lower PSA, smaller tumours, less involvement of surgical margins and/or extra-prostatic tissues with cancer and less diffuse involvement of the prostate by HGPIN compared with the subjects in the control group.
[52]	Prostate cancer	2009	Germany	Intervention	18	15 mg once daily	PSA response, clinical progression, quality of life, pain relief	Negative	No clinically relevant benefits (analgesic use and quality of life) were shown for patients with progressive hormone-refractory prostate cancer.
[48]	Prostate cancer	2007	USA	Intervention	46	15 mg twice daily	PSA response, tumour response, toxicity	Negative	Lycopene did not appear effective for treating androgen-independent prostate cancer.

[45]	Prostate cancer	2006	USA	Intervention	36	15, 30, 45, 60, 90 and 120 mg once daily	PSA response, pharmacokinetics, toxicity	Negative	Lycopene supplementation in men with biochemically relapsed prostate cancer did not result in any discernible response in serum PSA.
[25]	Prostate cancer	2002	USA	Intervention	26	15 mg twice daily	PSA response, tumour stage, Gleason score and extent of HGPIN	Positive	Lycopene intervention resulted in smaller tumours, organ-confined disease without involvement of surgical margins or extra-prostatic tissues and focal involvement of the prostate with HGPIN. The mean plasma PSA levels were lower in the intervention group than in the control group.

References:

[19] Tang, Y.; Parmakhtiar, B.; Simoneau, A.R.; Xie, J.; Fruehauf, J.; Lilly, M.; Zi, X. Lycopene enhances docetaxel's effect in castration-resistant prostate cancer associated with insulin-like growth factor I receptor levels. *Neoplasia* **2011**, *13*, 108–119. https://doi.org/10.1593/neo.101092.

[25] Kristal, A.R.; Till, C.; Platz, E.A.; Song, X.; King, I.B.; Neuhouser, M.L.; Ambrosone, C.B.; Thompson, I.M. Serum lycopene concentration and prostate cancer risk: Results from the Prostate Cancer Prevention Trial. *Cancer Epidemiol. Biomark. Prev.* **2011**, *20*, 638–646. https://doi.org/10.1158/1055-9965.EPI-10-1221.

[30] Morgia, G.; Voce, S.; Palmieri, F.; Gentile, M.; Iapicca, G.; Giannantoni, A.; Blefari, F.; Carini, M.; Vespasiani, G.; Santelli, G.; et al. Association between selenium and lycopene supplementation and incidence of prostate cancer: Results from the post-hoc analysis of the procomb trial. *Phytomedicine* **2017**, *34*, 1–5. https://doi.org/10.1016/j.phymed.2017.06.008.

[33] Vogt, T.M.; Mayne, S.T.; Graubard, B.I.; Swanson, C.A.; Sowell, A.L.; Schoenberg, J.B.; Swanson, G.M.; Greenberg, R.S.; Hoover, R.N.; Hayes, R.B.; et al. Serum lycopene, other serum carotenoids, and risk of prostate cancer in US Blacks and Whites. *Am. J. Epidemiol.* **2002**, *155*, 1023–1032. https://doi.org/10.1093/aje/155.11.1023.

45] Rowles, J.L.; Smith, J.W.; Applegate, C.C.; Miller, R.J.; Wallig, M.A.; Kaur, A.; Sarol, J.N.; Musaad, S.; Clinton, S.K.; O'Brien, W.D.; et al. Dietary Tomato or Lycopene Do Not Reduce Castration-Resistant Prostate Cancer Progression in a Murine Model. *J. Nutr.* **2020**, *150*, 1808–1817. https://doi.org/10.1093/jn/nxaa107.

[48] Magbanua, M.J.M.; Roy, R.; Sosa, E.V.; Weinberg, V.; Federman, S.; Mattie, M.D.; Hughes-Fulford, M.; Simko, J.; Shinohara, K.; Haqq, C.M.; et al. Gene expression and biological pathways in tissue of men with prostate cancer in a randomized clinical trial of lycopene and fish oil supplementation. *PLoS ONE* **2011**, *6*, e24004. https://doi.org/10.1371/journal.pone.0024004.

[49] van Breemen, R.B.; Sharifi, R.; Viana, M.; Pajkovic, N.; Zhu, D.; Yuan, L.; Yang, Y.; Bowen, P.E.; Stacewicz-Sapuntzakis, M. Antioxidant effects of lycopene in African American men with prostate cancer or benign prostate hyperplasia: A randomized, controlled trial. *Cancer Prev. Res.* **2011**, *4*, 711–718. https://doi.org/10.1158/1940-6207.CAPR-10-0288.

[50] Clark, P.E.; Hall, M.C.; Borden, L.S.J.; Miller, A.A.; Hu, J.J.; Lee, W.R.; Stindt, D.; D'Agostino, R.J.; Lovato, J.; Harmon, M.; et al. Phase I-II prospective dose-escalating trial of lycopene in patients with biochemical relapse of prostate cancer after definitive local therapy. *Urology* **2006**, *67*, 1257–1261. https://doi.org/10.1016/j.urology.2005.12.035.

[51] Rao, A.V.; Fleshner, N.; Agarwal, S. Serum and tissue lycopene and biomarkers of oxidation in prostate cancer patients: A case-control study. *Nutr. Cancer* **1999**, *33*, 159–164. https://doi.org/10.1207/S15327914NC330207.

[52] Giovannucci, E.; Rimm, E.B.; Liu, Y.; Stampfer, M.J.; Willett, W.C. A prospective study of tomato products, lycopene, and prostate cancer risk. *J. Natl. Cancer Inst.* **2002**, *94*, 391–398. https://doi.org/10.1093/jnci/94.5.391.
