# Supplementary material for: The Anti-Cancer Activity of Lycopene: A Systematic Review of Human and Animal Studies
Source: Nutrients. 2022 Dec 3;14(23):5152. doi: 10.3390/nu14235152 (PMC9741066; doi:10.3390/nu14235152)
Supplement: Supplementary file 1 [file nutrients-14-05152-s001.zip › Supplement 4.pdf]

Supplement 4. Overview of the analysed animal-based studies

Reference in paper	Cancer	Year	Animal	Intervention	Endpoint	Outcome	Finding
[68]	Breast cancer	2011	Rats	Carcinogen to induce cancer + lycopene +/- genistein supplementation for 20 weeks	Cancer incidence, tumour mass and volume	Positive	70% reduction of mammary cancer incidence by lycopene and 40% by genistein + lycopene; 48% and 67% reduction of tumour weight, respectively; and 18% and 65% mean tumour volumes, respectively
[69]	Colon cancer	2012	Mice	Lycopene–fish oil supplementation	Tumour growth and progression	Positive	Lycopene and fish oil synergistically inhibited the growth of colon cancer in tumour-bearing mice. Underlying mechanism of prevention: ↑ expression of p21(cip1/waf1) and p27(kip1) (cell cycle inhibitors); inhibition of cell division: ↓ of PCNA, β-catenin, cyclin D1 and C-myc proteins; inhibition of progression: ↓ MMP-7, MMP-9, COX-2 and PGE2
[63]	Colon cancer	2011	Mice	Lycopene supplementation for 5 weeks	Growth and progression of colorectal cancer	Positive	Lycopene could act as a chemopreventive agent against the growth and progression of colorectal cancer.
[81]	Oesophageal cancer	2020	Rats	Normal diets containing different concentrations of lycopene for 25 weeks	Incidence of cancer and antioxidant activity	Positive	The incidence of tumours in the rats treated with lycopene was significantly lower than in the controls. The study confirmed the possible anti-inflammatory and pro-apoptotic mechanisms.
[73]	Gastric cancer	2013	Rats	Carcinogen to induce cancer + lycopene supplementation (at increasing doses) for 26 weeks	Expression of oxidative stress markers	Positive	Lycopene may play an important role in protecting against oxidative stress and gastric carcinogenesis by enhancing antioxidant enzyme activities and immunity activities.
[83]	Gastric cancer	2011	Rats	Carcinogen to induce cancer + lycopene supplementation at different doses for 21 weeks	Expression of oxidative injury and immunity activities	Positive	Lycopene upregulated the redox status and immunity activities and finally decreased the risk of gastric cancer.
[93]	Gastric cancer	2005	Rats	Lycopene +/- S-allylcysteine +/- carcinogen	Incidence of gastric cancer, redox status markers	Positive	S-allylcysteine and lycopene significantly suppressed the development of gastric cancer, but the supplements given in combination were more effective.
[71]	Gastric cancer	2002	Rats	Lycopene +/- carcinogen	Incidence of gastric cancer, redox status markers	Positive	Lycopene may exert inhibitory effects by modulating the oxidant and antioxidant status in the gastric mucosa.
[72]	Gastric cancer	2006	Ferrets	Lycopene at low and high doses +/- carcinogen	Changes in protein levels of cell proliferation and apoptosis markers	Positive	Lycopene may prevent smoke exposure-induced cell proliferation and apoptosis in the gastric mucosa.
[60]	Hepatocellula r cancer (HCC)	2001	Rats	Lycopene supplementation for 70 weeks	Volume of HCC; concentration of copper, iron and zinc; expression of GST-P (a marker for preneoplastic or neoplastic lesions in chemical hepatocarcinogenesis)	Negative	Long-term administration of lycopene did not reduce the risk of hepatocarcinogenesis.

[88]	HCC	2015	Mice	Lycopene supplementation for 12 weeks +/- carcinogen	Incidence of HCC; levels of hypoxia, angiogenesis and metastatic markers	Positive	Lycopene supplementation may counteract HCC progression and/or protect against disease onset.
[62]	HCC	2008	Mice	Lycopene supplementation at low and high doses for 12 weeks	Incidence of metastases	Positive	Lycopene supplementation reduces tumour metastasis and suggests that such an action is associated with attenuation of tumour invasion, proliferation and angiogenesis.
[57]	Lung and hepatocellular cancer	2016	Ferrets	Carcinogen to induce cancer + lycopene supplementation (two doses) for 26 weeks	Incidence of cancers and mortality	Positive	Lycopene could provide potential benefits against smoke carcinogen-induced pulmonary and hepatic cancers.
[85]	Lung cancer	2000	Mice	Carcinogen to induce cancer +/- lycopene supplementation (at two doses) for 21 weeks	Incidence of lung cancer	Positive	Lycopene may have potential as a chemopreventive agent against carcinogenesis in the male lung.
[79]	Lung cancer	2019	Mice	Anti-mouse PD-1 antibodies 3 days apart for 4 times, and daily lycopene	Influence of lycopene on the effect of anti-PD-1 treatment on lung cancer	Positive	Lycopene could be used as a potential adjuvant drug to synergistically improve the efficiency of anti-PD-1 therapy.
[80]	Lung cancer	2006	Mice	Lycopene–D-arginine supplementation	Incidence of lung cancer	Positive	Post-treatment with lycopene and D-arginine prevented lung cancer in animals.
[91]	Lung cancer	2003	Ferrets	Lycopene supplementation at low and high doses for 9 weeks	Incidence of lung cancer, levels of proliferation and apoptosis markers	Positive	IGFBP-3 upregulation and BAD phosphorylation downregulation, which promote apoptosis and inhibit cell proliferation, may mediate the protective effects of lycopene against smoke-induced lung carcinogenesis in ferrets.
[94]	Ovarian cancer	2018	Hens	Lycopene supplementation (at two doses) for 12 months	Incidence of ovarian cancer, levels of oxidative stress markers and molecular mode of action	Positive	Lycopene has potential in the chemoprevention of ovarian cancer through antioxidant and anti-inflammatory mechanisms.
[39]	Prostate cancer	2020	Mice	(1) Lycopene-rich diet from the beginning or starting after castration<br>(2) Lycopene supplementation +/- lycopene-rich diet	Incidence and growth of castration-resistant prostate cancer	Negative	Tomato components had no significant impact on the emergence of castrate-resistant prostate cancer.
[15]	Prostate cancer	2010	Mice	Lycopene-rich diet or lycopene supplementation for 20 weeks	Incidence of prostate cancer, levels of IGF-I and IGF-binding protein-3, mean oxidative DNA damage	Positive	Lycopene supplementation (in contrast to lycopene-rich diet, such as tomato paste) plays a chemopreventive role in prostate cancer through oxidative DNA damage reduction.
[36]	Prostate cancer	2004	Rats	Lycopene–vitamin E supplementation for 4 weeks	Tumour volume, lycopene serum levels and molecular mode of action	Positive	Lycopene and vitamin E contribute to prostate cancer risk reduction by interfering with internal autocrine or paracrine loops of sex steroid hormone and growth factor activation/synthesis and signalling in the prostate.
[16]	Prostate cancer	2015	Mice	Low lycopene-containing tomato powder diet	Incidence of prostate cancer	Negative	A low-lycopene tomato powder intervention failed to reduce carcinogenesis.

[28]	Prostate cancer	2006	Mice	Lycopene +/- vitamin E supplementation daily	Tumour growth and prostate-specific antigen levels	Inconclusive	Lycopene alone did not reduce the tumour volume, but combined with vitamin E, it reduced the growth of prostate tumours by 73% and increased the median survival time by 40%.
[22]	Prostate cancer	2005	Mice	Lycopene supplementation (at two doses) for 8 weeks	Tumour growth	Positive	Lycopene may inhibit the growth specifically of androgen-independent prostate cancers.
[56]	Prostate cancer	2011	Mice	Lycopene +/- docetaxel (at two doses)	Cell proliferation and apoptosis	Positive	Docetaxel + lycopene supplement resulted in increases in apoptotic cells approximately 98% and 392% more than those resulting from docetaxel and lycopene supplements alone, respectively.
[14]	Prostate cancer	2003	Rats	Lycopene-rich diet or lycopene supplementation + carcinogen	Prostate cancer-specific mortality, prostate cancer-free survival	Positive	Consumption of tomato powder but not lycopene inhibited prostate carcinogenesis, suggesting that tomato products contain compounds in addition to lycopene that modify prostate carcinogenesis.
[54]	Prostate cancer	2007	Rats	Lycopene-rich diet or lycopene supplementation	Lycopene-rich diet or lycopene supplementation	Positive	Lycopene in low and high doses insignificantly reduced the tumour weights by 7% and 18%, respectively, whereas tomato reduced the tumour weight by 34% ($p < 0.05$).
[35]	Prostate cancer	2001	Rats	Incidence of prostate cancer	Lycopene or curcumin and carcinogen	Negative	Neither lycopene nor curcumin can consistently prevent rat prostate carcinogenesis.
[27]	Prostate cancer	2011	Mice	Cancer growth	Low or high dose of lycopene (4 and 16 mg/kg) and a single dose of β-carotene (16 mg/kg) twice a week for 7 weeks	Positive	Both lycopene and β-carotene strongly inhibited tumour growth.
[34]	Prostate cancer	2004	Mice	Incidence of prostate cancer	Vitamin E, selenium and lycopene	Positive	Treatment of animals with the antioxidants resulted in a fourfold reduction in the incidence of prostate cancer compared with the untreated animals.
[58]	Renal cell cancer	2015	Rats	Lycopene supplementation (at two doses) for 18 months	Incidence of renal cell cancer	Positive	Dietary supplementation with lycopene attenuates the development of renal cell cancers.
[86]	skin cancers	2018	Mice	Lycopene supplementation for 5 days	Type B ultraviolet (UVB) radiation-induced carcinogenesis and modulation of mTORC2/AKT/FOXO3a signalling pathway	Positive	The mTORc2/AKT/FOXO3A axis plays a critical role in the anti-proliferative and pro-apoptotic effects of lycopene in UVB-induced photocarcinogenesis.

References

[14] Graff, R.E.; Pettersson, A.; Lis, R.T.; Ahearn, T.U.; Markt, S.C.; Wilson, K.M.; Rider, J.R.; Fiorentino, M.; Finn, S.; Kenfield, S.A.; et al. Dietary lycopene intake and risk of prostate cancer defined by ERG protein expression. *Am. J. Clin. Nutr.* **2016**, *103*, 851–860. https://doi.org/10.3945/ajcn.115.118703.
[15] Schwenke, C.; Ubrig, B.; Thürmann, P.; Eggersmann, C.; Roth, S. Lycopene for advanced hormone refractory prostate cancer: A prospective, open phase II pilot study. *J. Urol.* **2009**, *181*, 1098–1103. https://doi.org/10.1016/j.juro.2008.11.012.

[16] Kirsh, V.A.; Mayne, S.T.; Peters, U.; Chatterjee, N.; Leitzmann, M.F.; Dixon, L.B.; Urban, D.A.; Crawford, E.D.; Hayes, R.B. A prospective study of lycopene and tomato product intake and risk of prostate cancer. *Cancer Epidemiol. Biomark. Prev.* **2006**, *15*, 92–98. https://doi.org/10.1158/1055-9965.EPI-05-0563.
[22] Boileau, T.W.-M.; Liao, Z.; Kim, S.; Lemeshow, S.; Erdman, J.W.J.; Clinton, S.K. Prostate carcinogenesis in N-methyl-N-nitrosourea (NMU)-testosterone-treated rats fed tomato powder, lycopene, or energy-restricted diets. *J. Natl. Cancer Inst.* **2003**, *95*, 1578–1586. https://doi.org/10.1093/jnci/djg081.
[27] Vaishampayan, U.; Hussain, M.; Banerjee, M.; Seren, S.; Sarkar, F.H.; Fontana, J.; Forman, J.D.; Cher, M.L.; Powell, I.; Pontes, J.E.; et al. Lycopene and soy isoflavones in the treatment of prostate cancer. *Nutr. Cancer* **2007**, *59*, 1–7. https://doi.org/10.1080/01635580701413934.
[28] Gann, P.H.; Ma, J.; Giovannucci, E.; Willett, W.; Sacks, F.M.; Hennekens, C.H.; Stampfer, M.J. Lower prostate cancer risk in men with elevated plasma lycopene levels: Results of a prospective analysis. *Cancer Res.* **1999**, *59*, 1225–1230.
[34] Yang, C.-M.; Yen, Y.-T.; Huang, C.-S.; Hu, M.-L. Growth inhibitory efficacy of lycopene and β-carotene against androgen-independent prostate tumor cells xenografted in nude mice. *Mol. Nutr. Food Res.* **2011**, *55*, 606–612. https://doi.org/10.1002/mnfr.201000308.
[35] Limpens, J.; Schröder, F.H.; de Ridder, C.M.A.; Bolder, C.A.; Wildhagen, M.F.; Obermüller-Jevic, U.C.; Krämer, K.; van Weerden, W.M. Combined lycopene and vitamin E treatment suppresses the growth of PC-346C human prostate cancer cells in nude mice. *J. Nutr.* **2006**, *136*, 1287–1293. https://doi.org/10.1093/jn/136.5.1287.
[36] Bowen, P.; Chen, L.; Stacewicz-Sapuntzakis, M.; Duncan, C.; Sharifi, R.; Ghosh, L.; Kim, H.-S.; Christov-Tzelkov, K.; van Breemen, R. Tomato sauce supplementation and prostate cancer: Lycopene accumulation and modulation of biomarkers of carcinogenesis. *Exp. Biol. Med.* **2002**, *227*, 886–893. https://doi.org/10.1177/153537020222701008.
[39] Chan, J.M.; Weinberg, V.; Magbanua, M.J.; Sosa, E.; Simko, J.; Shinohara, K.; Federman, S.; Mattie, M.; Hughes-Fulford, M.; Haqq, C.; et al. Nutritional supplements, COX-2 and IGF-1 expression in men on active surveillance for prostate cancer. *Cancer Causes Control* **2011**, *22*, 141–150. https://doi.org/10.1007/s10552-010-9684-5.
[54] Kucuk, O.; Sarkar, F.H.; Djuric, Z.; Sakr, W.; Pollak, M.N.; Khachik, F.; Banerjee, M.; Bertram, J.S.; Wood, D.P. Effects of lycopene supplementation in patients with localized prostate cancer. *Exp. Biol. Med.* **2002**, *227*, 881–885. https://doi.org/10.1177/153537020222701007.
[56] Ansari, M.S.; Gupta, N.P. A comparison of lycopene and orchidectomy vs orchidectomy alone in the management of advanced prostate cancer. *BJU Int.* **2003**, *92*, 375–378; discussion 378. https://doi.org/10.1046/j.1464-410x.2003.04370.x.
[57] Aizawa, K.; Liu, C.; Tang, S.; Veeramachaneni, S.; Hu, K.-Q.; Smith, D.E.; Wang, X.-D. Tobacco carcinogen induces both lung cancer and non-alcoholic steatohepatitis and hepatocellular carcinomas in ferrets which can be attenuated by lycopene supplementation. *Int. J. Cancer* **2016**, *139*, 1171–1181. https://doi.org/10.1002/ijc.30161.
[58] Sahin, K.; Cross, B.; Sahin, N.; Ciccone, K.; Suleiman, S.; Osunkoya, A.O.; Master, V.; Harris, W.; Carthon, B.; Mohammad, R.; et al. Lycopene in the prevention of renal cell cancer in the TSC2 mutant Eker rat model. *Arch. Biochem. Biophys.* **2015**, *572*, 36–39. https://doi.org/10.1016/j.abb.2015.01.006.
[60] Watanabe, S.; Kitade, Y.; Masaki, T.; Nishioka, M.; Satoh, K.; Nishino, H. Effects of lycopene and Sho-saiko-to on hepatocarcinogenesis in a rat model of spontaneous liver cancer. *Nutr. Cancer* **2001**, *39*, 96–101. https://doi.org/10.1207/S15327914nc391_13.
[62] Huang, C.-S.; Liao, J.-W.; Hu, M.-L. Lycopene inhibits experimental metastasis of human hepatoma SK-Hep-1 cells in athymic nude mice. *J. Nutr.* **2008**, *138*, 538–543. https://doi.org/10.1093/jn/138.3.538.
[63] Tang, F.-Y.; Pai, M.-H.; Wang, X.-D. Consumption of lycopene inhibits the growth and progression of colon cancer in a mouse xenograft model. *J. Agric. Food Chem.* **2011**, *59*, 9011–9021. https://doi.org/10.1021/jf2017644.
[68] Sahin, K.; Tuzcu, M.; Sahin, N.; Akdemir, F.; Ozercan, I.; Bayraktar, S.; Kucuk, O. Inhibitory effects of combination of lycopene and genistein on 7,12- dimethyl benz(a)anthracene-induced breast cancer in rats. *Nutr. Cancer* **2011**, *63*, 1279–1286. https://doi.org/10.1080/01635581.2011.606955.
[69] Tang, F.-Y.; Pai, M.-H.; Kuo, Y.-H.; Wang, X.-D. Concomitant consumption of lycopene and fish oil inhibits tumor growth and progression in a mouse xenograft model of colon cancer. *Mol. Nutr. Food Res.* **2012**, *56*, 1520–1531. https://doi.org/10.1002/mnfr.201200098.
[71] Velmurugan, B.; Bhuvaneswari, V.; Burra, U.K.; Nagini, S. Prevention of N-methyl-N′-nitro-N-nitrosoguanidine and saturated sodium chloride-induced gastric carcinogenesis in Wistar rats by lycopene. *Eur. J. Cancer Prev.* **2002**, *11*, 19–26. https://doi.org/10.1097/00008469-200202000-00004.
[72] Liu, C.; Russell, R.M.; Wang, X.-D. Lycopene supplementation prevents smoke-induced changes in p53, p53 phosphorylation, cell proliferation, and apoptosis in the gastric mucosa of ferrets. *J. Nutr.* **2006**, *136*, 106–111. https://doi.org/10.1093/jn/136.1.106.

[73] Xu, Y.F.; Zhou, S.K.; Feng, X.J.; Jiang, L.X. Modulatory effect of lycopene on oxidative injury, related proteins and gene expression in gastric cancer tissue. *J. Food Agric. Environ.* **2013**, *11*, 511–515.
[79] Jiang, X.; Wu, H.; Zhao, W.; Ding, X.; You, Q.; Zhu, F.; Qian, M.; Yu, P. Lycopene improves the efficiency of anti-PD-1 therapy via activating IFN signaling of lung cancer cells. *Cancer Cell Int.* **2019**, *19*, 68. https://doi.org/10.1186/s12935-019-0789-y.
[80] Radhakrishnan, V.K.; Ravikumar, V.; Shivashangari, K.; Sattu, K.; Devaki, T. Chemopreventive Role of Lycopene and D-arginine in Benzo(a) Pyrene Induced Lung Cancer with Reference to Lipid Peroxidation, Antioxidant and Tumor Marker Enzymes. *Int. J. Cancer Res.* **2006**, *2*, 224–233. https://doi.org/10.3923/ijcr.2006.224.233.
[81]Cui, L.; Xu, F.; Wu, K.; Li, L.; Qiao, T.; Li, Z.; Chen, T.; Sun, C. Anticancer effects and possible mechanisms of lycopene intervention on N-methylbenzylnitrosamine induced esophageal cancer in F344 rats based on PPARγ(1). *Eur. J. Pharmacol.* **2020**, *881*, 173230. https://doi.org/10.1016/j.ejphar.2020.173230.
[83] Luo, C.; Wu, X.-G. Lycopene enhances antioxidant enzyme activities and immunity function in N-methyl-N′-nitro-N-nitrosoguanidine-enduced gastric cancer rats. *Int. J. Mol. Sci.* **2011**, *12*, 3340–3351. https://doi.org/10.3390/ijms12053340.
[85] Kim, D.J.; Takasuka, N.; Nishino, H.; Tsuda, H. Chemoprevention of lung cancer by lycopene. *Biofactors* **2000**, *13*, 95–102. https://doi.org/10.1002/biof.5520130116.
[86] Chen, P.; Xu, S.; Qu, J. Lycopene Protects Keratinocytes Against UVB Radiation-Induced Carcinogenesis via Negative Regulation of FOXO3a Through the mTORC2/AKT Signaling Pathway. *J. Cell. Biochem.* **2018**, *119*, 366–377. https://doi.org/10.1002/jcb.26189.
[88] Bhatia, N.; Gupta, P.; Singh, B.; Koul, A. Lycopene Enriched Tomato Extract Inhibits Hypoxia, Angiogenesis, and Metastatic Markers in early Stage N-Nitrosodiethylamine Induced Hepatocellular Carcinoma. *Nutr. Cancer* **2015**, *67*, 1268–1275. https://doi.org/10.1080/01635581.2015.1087040.
[91] Liu, C.; Lian, F.; Smith, D.E.; Russell, R.M.; Wang, X.-D. Lycopene supplementation inhibits lung squamous metaplasia and induces apoptosis via up-regulating insulin-like growth factor-binding protein 3 in cigarette smoke-exposed ferrets. *Cancer Res.* **2003**, *63*, 3138–3144.
[93] Velmurugan, B.; Nagini, S. Combination chemoprevention of experimental gastric carcinogenesis by s-allylcysteine and lycopene: Modulatory effects on glutathione redox cycle antioxidants. *J. Med. Food* **2005**, *8*, 494–501. https://doi.org/10.1089/jmf.2005.8.494.
[94] Sahin, K.; Yenice, E.; Tuzcu, M.; Orhan, C.; Mizrak, C.; Ozercan, I.H.; Sahin, N.; Yilmaz, B.; Bilir, B.; Ozpolat, B.; et al. Lycopene Protects Against Spontaneous Ovarian Cancer Formation in Laying Hens. *J. Cancer Prev.* **2018**, *23*, 25–36. https://doi.org/10.15430/JCP.2018.23.1.25.
