# Supplementary material for: The Anti-Cancer Activity of Lycopene: A Systematic Review of Human and Animal Studies
Source: Nutrients. 2022 Dec 3;14(23):5152. doi: 10.3390/nu14235152 (PMC9741066; doi:10.3390/nu14235152)
Supplement: Supplementary file 1 [file nutrients-14-05152-s001.zip › Supplement 5.pdf]

Supplement 5. Overview of human studies on lycopene in cancer with endpoints other than outcomes of treatment or prevention

Reference in paper	Cancer	Year	Country	Study design	Population size	Lycopene supplementation or source	Endpoint	Outcome	Finding
[3]	Any cancer	1998	Canada	Intervention	19	Tomato juice, spaghetti sauce and tomato oleoresin for a period of one week each	Uptake and antioxidant properties of lycopene	Positive	Lycopene dietary supplementation increased the serum lycopene level and diminished the amounts of serum thiobarbituric acid-reactive substances.
[75]	Breast cancer	2012	Italy	Intervention	71	Resveratrol, lycopene, vitamin C and anthocyanin (Ixor®) oral supplements at doses of 2 tablets daily from 10 days before radiotherapy to 10 days after	Reduction of skin toxicity during RTH	Positive	The protective effects of resveratrol, lycopene, vitamin C and anthocyanin (Ixor®) were detected more in patients with the planning target volume less than 500 ml and in patients undergoing adjuvant chemotherapy with anthracyclines and taxanes.
[65]	Breast cancer	2008	Netherlands	Intervention	60	Lycopene 30 mg daily for 2 months	Serum levels of total IGF-I	Inconclusive	Lycopene supplementation has no effect on serum total IGF-I in the overall study population but showed beneficial effects in high-risk healthy women, although not in breast cancer survivors.
[76]	Colon cancer	2007	Israel	Intervention	56	Dietary tomato lycopene extract supplementation for a few days to a few weeks	Serum levels of the IGF system components	Positive	Lycopene extract supplementation decreases IGF-I.
[61]	Colorectal cancer	2007	Netherlands	Intervention	71	30 mg of tomato-derived lycopene daily for 8 weeks	Serum concentrations of total IGF-I, IGF-II, IGFBP-1, IGFBP-2 and IGFBP-3	Positive	Lycopene supplementation may increase circulating IGFBP-1 and IGFBP-2 concentrations.
[90]	Colorectal cancer	2021	Italy	Intervention	28	20 mg lactolycopene daily for the entire duration of the treatment with panitumumab	Skin toxicity, frequency of tetracycline administration, malondialdehyde production	Positive	The effectiveness of lycopene in reducing skin toxicity and tetracycline administration and protecting tissues from oxidative stress was confirmed in patients treated with panitumumab.
[13]	Prostate cancer	2019	USA	Intervention	47	0, 1 or 2 cans of tomato-soy juice daily	Changes in the plasma and prostate concentrations of lycopene depending on single nucleotide polymorphism (SNP) in or near known or putative carotenoid metabolism genes	Positive	The SNPs in β-carotene oxygenase 1 are associated with plasma lycopene responses to a tomato-soy juice intervention.

[43]	Prostate cancer	2011	USA	Intervention	105	30 mg daily as a tomato oleoresin or placebo for 21 days prior to prostate biopsy	Lycopene levels in blood and prostate tissue and levels of markers of oxidative stress	Inconclusive	The mean lycopene concentration increased in plasma and in prostate tissue, but no significant changes in the DNA oxidation and the lipid peroxidation products were observed in prostate tissue and plasma.
[42]	Prostate cancer	2011	USA	Intervention	84	Lycopene 2 x 15 mg daily for 3 months	Gene expression in prostate tissue	Inconclusive	Lycopene supplementation resulted in the modulation of androgen and estrogen metabolism and of Nrf2 or Nrf2-mediated oxidative stress response.
[32]	Prostate cancer	2011	USA	Intervention	69	Lycopene 30 mg daily	IGF-1 and COX-2 expression	Negative	Lycopene or fish oil supplementation did not significantly change IGF-1 and COX-2 gene expression in the normal prostate microenvironment in men with low-burden prostate cancer.
[29]	Prostate cancer	2002	USA	Intervention	32	Lycopene 30 mg daily	Serum and prostate lycopene, prostate-specific antigen concentrations, 8-OHdG staining and apoptosis	Positive	Supplementation causes significant lycopene uptake into prostate tissue and a reduction in DNA damage in both leukocyte and prostate tissues.

[3] Rao, A.V.; Waseem, Z.; Agarwal, S. Lycopene content of tomatoes and tomato products and their contribution to dietary lycopene. *Food Res. Int.* **1998**, *31*, 737–741. https://doi.org/10.1016/S0963-9969(99)00053-8.

[13] Zhuang, E.; Uchio, E.; Lilly, M.; Zi, X.; Fruehauf, J.P. A phase II study of docetaxel plus lycopene in metastatic castrate resistant prostate cancer. *Biomed. Pharmacother.* **2021**, *143*, 112226. https://doi.org/10.1016/j.biopha.2021.112226.

[29] Fraser, G.E.; Jacobsen, B.K.; Knutsen, S.F.; Mashchak, A.; Lloren, J.I. Tomato consumption and intake of lycopene as predictors of the incidence of prostate cancer: The Adventist Health Study-2. *Cancer Causes Control* **2020**, *31*, 341–351. https://doi.org/10.1007/s10552-020-01279-z.

[32] Kucuk, O.; Sarkar, F.H.; Sakr, W.; Khachik, F.; Djuric, Z.; Banerjee, M.; Pollak, M.N.; Bertram, J.S.; Wood, D.P. Lycopene in the treatment of prostate cancer. *Pure Appl. Chem.* **2002**, *74*, 1443–1450, doi:doi:10.1351/pac200274081443.

[42] Siler, U.; Barella, L.; Spitzer, V.; Schnorr, J.; Lein, M.; Goralczyk, R.; Wertz, K. Lycopene and vitamin E interfere with autocrine/paracrine loops in the Dunning prostate cancer model. *FASEB J. Off. Publ. Fed. Am. Soc. Exp. Biol.* **2004**, *18*, 1019–1021. https://doi.org/10.1096/fj.03-1116fje.

[43] Lu, Q.Y.; Hung, J.C.; Heber, D.; Go, V.L.; Reuter, V.E.; Cordon-Cardo, C.; Scher, H.I.; Marshall, J.R.; Zhang, Z.F. Inverse associations between plasma lycopene and other carotenoids and prostate cancer. *Cancer Epidemiol. Biomark. Prev.* **2001**, *10*, 749–756.

[61] Vrieling, A.; Voskuil, D.W.; Bonfrer, J.M.; Korse, C.M.; van Doorn, J.; Cats, A.; Depla, A.C.; Timmer, R.; Witteman, B.J.; van Leeuwen, F.E.; et al. Lycopene supplementation elevates circulating insulin-like growth factor binding protein-1 and -2 concentrations in persons at greater risk of colorectal cancer. *Am. J. Clin. Nutr.* **2007**, *86*, 1456–1462. https://doi.org/10.1093/ajcn/86.5.1456.

[65] Voskuil, D.W.; Vrieling, A.; Korse, C.M.; Beijnen, J.H.; Bonfrer, J.M.G.; van Doorn, J.; Kaas, R.; Oldenburg, H.S.A.; Russell, N.S.; Rutgers, E.J.T.; et al. Effects of lycopene on the insulin-like growth factor (IGF) system in premenopausal breast cancer survivors and women at high familial breast cancer risk. *Nutr. Cancer* **2008**, *60*, 342–353. https://doi.org/10.1080/01635580701861777.

[75] Di Franco, R.; Calvanese, M.; Murino, P.; Manzo, R.; Guida, C.; Di Gennaro, D.; Anania, C.; Ravo, V. Skin toxicity from external beam radiation therapy in breast cancer patients: protective effects of Resveratrol, Lycopene, Vitamin C and anthocianin (Ixor®). *Radiat. Oncol.* **2012**, *7*, 12. https://doi.org/10.1186/1748-717X-7-12.
[76] Walfisch, S.; Walfisch, Y.; Kirilov, E.; Linde, N.; Mnitentag, H.; Agbaria, R.; Sharoni, Y.; Levy, J. Tomato lycopene extract supplementation decreases insulin-like growth factor-I levels in colon cancer patients. *Eur. J. Cancer Prev.* **2007**, *16*, 298–303. https://doi.org/10.1097/01.cej.0000236251.09232.7b.
[90] Moroni, M.; Pirovano, M.; Brugnatelli, S.; Zucca, M.; Morreale, M.; Rizzo, V.; Ferrari, A.; Tinelli, C.; De Silvestri, A.; Meregalli, M.; et al. Lycopene minimizes skin toxicity and oxidative stress in patients treated with panitumumab-containing therapy for metastatic colorectal cancer. *J. Funct. Foods* **2021**, *83*, 104533. https://doi.org/10.1016/j.jff.2021.104533.
